# Early Sonographic Markers of Gestational Diabetes Mellitus: A Narrative Review of Placental, Fetal and Uterine Artery Findings

**DOI:** 10.3390/biomedicines14081846

**Published:** 2026-08-17

**Authors:** Andreea Fotă, Aida Petca

**Affiliations:** 1 Department of Obstetrics and Gynecology, “Carol Davila” University of Medicine and Pharmacy, 8 Eroii Sanitari Blvd., 050474 Bucharest, Romania; aida.petca@umfcd.ro; 2 Department of Obstetrics and Gynecology, CF2 Clinical Hospital, 63 Mărăști Blvd., 011464 Bucharest, Romania

**Keywords:** gestational diabetes mellitus, placenta, fetus, uterine artery, morphology, early pregnancy

## Abstract

**Background/Objectives:** Gestational diabetes mellitus (GDM) is one of the most common metabolic complications of pregnancy and is associated with adverse maternal and neonatal outcomes. Current screening strategies are generally performed during the end of the second trimester, limiting opportunities for early intervention. Increasing evidence suggests that placental and fetal ultrasound markers detectable in early pregnancy may reflect the pathophysiological changes preceding the clinical diagnosis of GDM. This narrative review aims to summarize the current evidence regarding first- and second-trimester placental and fetal ultrasound markers associated with the subsequent development of GDM. **Methods:** A narrative review of the literature was conducted focusing on studies evaluating ultrasound markers of placental morphology, placental vascularization and function, uterine artery Doppler indices, placental volume, placental thickness, and fetal biometric and functional parameters in relation to later GDM diagnosis. Twenty-two studies were identified and analyzed to assess their clinical utility and predictive value. **Results:** Increasing evidence suggests that placental and fetal ultrasound markers detectable in early pregnancy may reflect pathophysiological changes preceding the clinical diagnosis of GDM. Three-dimensional power Doppler, particularly the vascularization–flow index, has demonstrated reduced placental vascularization in pregnancies subsequently complicated by GDM. Placental elastography and artificial intelligence-based radiomic analysis also show promising predictive potential. Uterine artery Doppler has demonstrated limited and inconsistent value. Fetal findings suggest that conventional biometry has limited predictive utility, whereas fetal growth velocity and markers of adiposity, including anterior abdominal wall thickness and abdominal overgrowth, more consistently identify early adaptations associated with later GDM. However, substantial methodological heterogeneity limits interpretation. **Conclusions:** Early placental and fetal ultrasound markers represent promising non-invasive tools for the early identification of pregnancies at risk for GDM. Further large-scale prospective studies are required to validate their predictive performance and establish standardized assessment protocols. Integrating maternal clinical characteristics and biochemical parameters may enhance future screening strategies for GDM.

## 1. Introduction

Gestational diabetes mellitus (GDM) is a common pregnancy-related metabolic complication, defined as glucose intolerance first diagnosed during pregnancy [1]. Its prevalence has been on a substantial rise over recent decades, driven by increasing rates of maternal obesity, sedentary lifestyles, advanced maternal age and changing diagnostic screening strategies [2,3]. Recent estimates place GDM at approximately 14% of pregnancies globally using the International Association of Diabetes in Pregnancy Study Group’s (IADPSG) Criteria [4], but prevalence varies greatly across populations and diagnostic criteria.

On that note, the International Association of Diabetes and Pregnancy Study Groups, the American Diabetes Association (ADA), and the World Health Organization (WHO) endorse a one-step approach based on findings from the Hyperglycemia and Adverse Pregnancy Outcome (HAPO) study [5]. The aforementioned strategy consists of a 75-g oral glucose tolerance test (OGTT) performed after fasting, between 24 and 28 weeks of gestation, with plasma glucose measured at fasting, 1 h, and 2 h. GDM is diagnosed when one or more glucose values meet or exceed the established thresholds: fasting ≥ 92 mg/dL (5.1 mmol/L), 1 h ≥ 180 mg/dL (10.0 mmol/L), or 2 h ≥ 153 mg/dL (8.5 mmol/L). The adoption of the IADPSG criteria has substantially increased the reported prevalence of GDM, primarily because only one abnormal value is required for diagnosis.

In contrast, the American College of Obstetricians and Gynecologists (ACOG) and the National Institute of Health (NIH) continue to recommend a two-step screening strategy [6]. This approach begins with a non-fasting 50 g glucose challenge test, followed by a diagnostic 100 g, 3-h OGTT, if the 1-h plasma glucose exceeds the screening threshold.

Ultrasonography plays a central role in the evaluation and surveillance of pregnancies complicated by gestational diabetes mellitus. Besides confirming fetal growth and gestational age, ultrasound enables the detection of structural and functional changes associated with maternal hyperglycemia, including excessive or insufficient fetal growth [7,8], altered body composition [9], oligo- or polyhydramnios [10], and fetal cardiac remodeling [11,12]. These findings provide valuable information regarding fetal metabolic status, facilitate risk stratification, and guide obstetric management, including the frequency of antenatal surveillance and the timing and mode of delivery. Ultrasound has become an indispensable adjunct to glycemic monitoring in pregnancies affected by GDM, because many sonographic abnormalities correlate with the degree and duration of maternal hyperglycemia.

Increasing evidence suggests that the effects of maternal hyperglycemia on the fetus and placenta begin well before routine biochemical screening [13]. Lately, attention has been directed toward the possibility of earlier identification of women at risk. Application of the IADPSG one-step approach using a 75-g OGTT during the first trimester has been investigated as a potential strategy for earlier diagnosis, supporting the concept that metabolic abnormalities may be identifiable before the conventional screening window [14]. However, biochemical testing in early pregnancy may not fully capture the early physiological changes associated with subsequent GDM. In this context, sonographic assessment of the placenta and fetus represents a potentially complementary, non-invasive approach for identifying structural, vascular, biomechanical, and growth-related alterations that may precede the clinical diagnosis of GDM. Subtle alterations in fetal growth patterns, body composition, placental morphology, cardiac structure and function, and maternal adipose tissue have been described during the first and second trimesters, preceding the clinical diagnosis of GDM [15,16,17]. As these features can be assessed during routine obstetric ultrasound examinations, they have emerged as potential imaging biomarkers for identifying pregnancies at increased risk of GDM. Early recognition of such sonographic markers could improve risk stratification, facilitate targeted metabolic screening and preventive interventions, and ultimately reduce adverse maternal and neonatal outcomes [13].

Therefore, the present review focuses on fetal, placental and uterine artery sonographic findings identified between 11 and 24 weeks of gestation that have been investigated as predictors of subsequent GDM.

## 2. Materials and Methods

**Figure 1:**
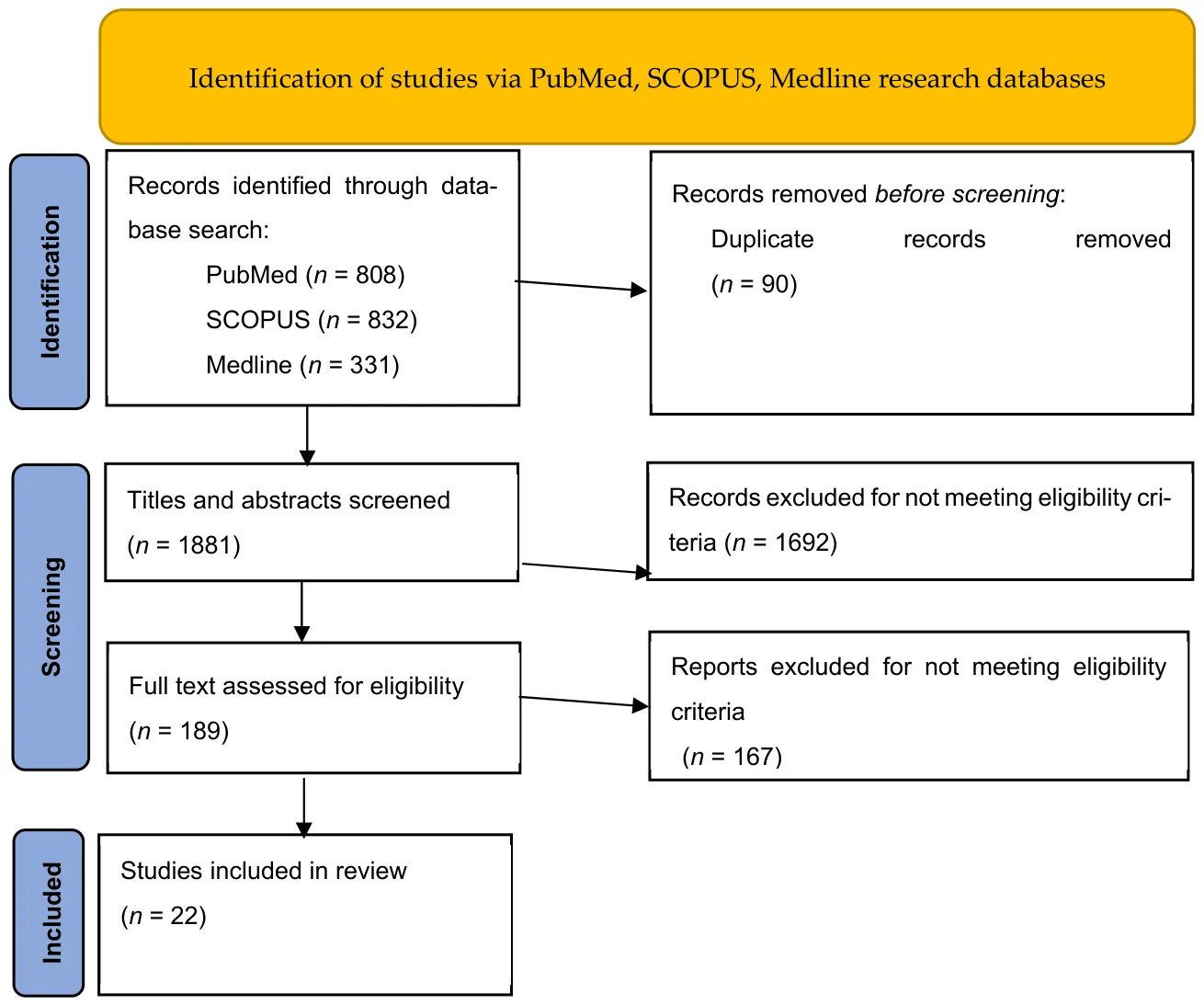
PRISMA-like flow diagram for the process of study identification.

This article is a narrative review of the specialty literature published in English. A comprehensive search was conducted using multiple databases, including PubMed, Scopus, Medline, with the scope of identifying studies related to ultrasonographic placental and fetal markers in relation to the development of gestational diabetes mellitus. Studies published from inception to 20 June 2026 were included (last search date: 20 June 2026). A detailed process is illustrated in Figure 1. Due to the narrative nature of the review, no formal risk-of-bias assessment, evidence grading, or quantitative synthesis was performed.

The search strategy included combinations of specific keywords occurring in either the titles or the abstract of the articles, such as “gestational diabetes mellitus”, “placenta”, “fetus”, “early pregnancy”, “ultrasonography”, “ultrasound”, and “Doppler” (Table 1).

Inclusion criteria encompassed clinical studies (randomized controlled trials, observational studies, and case series) on human participants; singleton pregnancies; placental and fetal ultrasound assessment performed between 11 weeks and 24 weeks of gestation, with GDM diagnosed later in pregnancy, according to guidelines.

Duplicates, articles lacking methodological clarity or data, animal studies, articles published in languages other than English, studies where GDM could not be separated from other pathologies, and systematic reviews and meta-analyses were also excluded. The systematic reviews and meta-analyses were included only in Section 4 to contextualize our findings within the existing literature.

## 3. Results

A total of 1971 studies were found. After the exclusion of 1949 studies, 22 studies that evaluated the aforementioned parameters regarding the placenta, fetus, and the uterine artery were included. Each of the following subchapters will provide a table summarizing the included articles, with emphasis on results highlighting the sonographic abnormalities.

### 3.1. Placenta

A total of seven studies were included, discussing placental morphology, vascularization and perfusion, as well as biomechanical properties and an integration of deep-learning into texture analysis. The characteristics of the studies are presented in Table 2.

#### 3.1.1. Placental Morphology, Vascularization, and Perfusion

Wong et al. [18] conducted a prospective case–control study including 155 singleton pregnancies at increased risk for GDM, out of which 31 who subsequently developed GDM, to investigate longitudinal changes in placental vascularization using three-dimensional power Doppler ultrasound. Placental vascular indices (vascularization index [VI], flow index [FI], and vascularization flow index [VFI]), placental volume, and uterine artery pulsatility index (PI) were measured during both the first trimester (11 + 0–13 + 6 weeks) and second trimester (21 + 0–23 + 6 weeks) routine ultrasounds, prior to routine GDM screening at 24–28 weeks. Women who subsequently developed GDM demonstrated significantly lower placental VI and VFI in both the first and second trimesters, whereas FI did not differ between groups. Placental volume was similar during the first trimester but became significantly bigger in pregnancies that later developed GDM during the second trimester. After adjustment for maternal age, BMI, and other confounders, first-trimester VFI (adjusted OR- odds ratio 0.76, 95% CI confidence interval 0.61–0.93), second-trimester VFI (adjusted OR 0.83, 95% CI 0.71–0.96), and second-trimester placental volume (adjusted OR 1.03, 95% CI 1.01–1.05) remained independently associated with subsequent GDM. The authors concluded that impaired placental vascularization precedes the clinical diagnosis of GDM and that VFI is a more sensitive early marker than placental volume, supporting the hypothesis that placental dysfunction begins during the first trimester.

In a prospective cohort of 141 singleton pregnancies, undergoing routine first-trimester screening, Han et al. [19] assessed placental volume and placental vascular indices, including the vascularization index, flow index, and vascularization–flow index. Participants subsequently underwent screening for GDM at 24–28 weeks, and 35 women were diagnosed with GDM. The former exhibited significantly lower placental volume and significantly reduced placental vascular indices during the first trimester compared with controls. Specifically, VI, FI, and VFI were all decreased in pregnancies that subsequently developed GDM. In logistic regression analysis, lower placental vascular indices remained significantly associated with GDM risk, with VFI demonstrating the strongest predictive value among the placental Doppler parameters evaluated. Furthermore, a prediction model incorporating maternal age, gravidity, placental volume, and VFI achieved an area under curve (AUC) of 0.76, outperforming individual placental markers alone.

Zhu et al. [20] evaluated 122 singleton pregnancies between 11 + 0 and 13 + 6 weeks of gestation using three-dimensional power Doppler ultrasound to assess placental volume and vascular indices. Among the cohort, 36 women subsequently developed GDM; those patients significantly lower placental volume and reduced placental vascularization indices during the first trimester. In multivariable analysis, placental volume, vascularization index, and flow index remained independent predictors of GDM. The authors subsequently constructed several predictive models. The best-performing model combined maternal clinical characteristics with ultrasound-derived placental parameters and achieved an AUC of 0.87. These findings suggest that first-trimester placental morphology and vascularization may contribute meaningful predictive information beyond established maternal risk factors.

Lavanya et al. [21] investigated placental thickness as an early sonographic marker of GDM in a prospective cross-sectional study of 139 singleton pregnancies undergoing ultrasound between 16 + 0 and 19 + 6 weeks gestation, with GDM diagnosed by oral glucose tolerance testing at 20–22 weeks. Placental thickness was measured at the placental cord insertion site and was significantly greater in women who subsequently developed GDM compared with those who remained normoglycemic (2.91 ± 0.66 cm vs. 2.35 ± 0.64 cm, *p* = 0.0001). Furthermore, placental thickness demonstrated a significant positive correlation with maternal random blood glucose levels in the overall cohort (r = 0.316, *p* = 0.0002), and the authors identified a placental thickness threshold of ≥2.7 cm as a significant predictor of GDM during the early second trimester.

#### 3.1.2. Placental Biomechanical Properties

Şengül et al. [22] aimed to investigate the placental strain ratio measured by real-time sonoelastography and maternal subcutaneous adipose tissue thickness on a population of 210 pregnant women who had been routinely screened between the 11th and 14th week of gestation. The diagnosis of GDM was further carried out between the 24th and 28th week of gestation, using a 75 g OGTT. Higher strain ratio reflects altered placental tissue stiffness/elastic properties. The prospective study demonstrated that placental biomechanical properties are altered as early as the first trimester, as placental strain ratio remained an independent predictor of GDM (OR = 3.664, 95% CI: 1.927–6.969, *p* < 0.001). The authors proposed a cutoff of >0.986 for early prediction of GDM, suggesting that early changes in placental tissue stiffness can precede the clinical manifestations of the glucose intolerance.

#### 3.1.3. Placental Texture Analysis

Peng et al. [23] conducted a multicenter retrospective study of 628 singleton pregnancies and investigated whether first-trimester placental ultrasound texture could predict subsequent GDM development. Ultrasound images were acquired at between 11 and 13 + 6 weeks of pregnancy and were characterized using 1289 radiomic features and 2048 deep-learning-derived features. Following feature fusion and selection using correlation, minimum redundancy–maximum relevance, and least absolute shrinkage and selection operator methods, a multimodal model demonstrated good predictive performance for subsequent GDM. However, the predictive signal was derived from a combination of quantitative imaging features rather than from a single reproducible radiomic parameter, and the biological significance of the selected features remains uncertain. Women who later developed GDM demonstrated placental imaging characteristics that could be distinguished from controls using machine-learning algorithms. The predictive features primarily reflected increased placental texture heterogeneity and deep learning-derived structural patterns, suggested to correspond to early placental morphological and vascular alterations, including reduced spiral artery branching, altered terminal vessel architecture, and focal hypoperfusion. The best-performing multimodal model, which integrated placental radiomic features, deep-learning representations, uterine artery Doppler parameters, and maternal clinical characteristics, achieved an AUC of 0.879 in the training cohort and 0.817 in external validation. However, the predictive signal was derived from a combination of quantitative imaging features rather than from a single reproducible radiomic parameter.

With a similar objective, Zhou et al. [24] conducted a multicenter retrospective study involving 415 singleton pregnancies, aiming to investigate whether artificial intelligence-based analysis could predict subsequent GDM. Placental imaging was performed between 11 and 13 weeks of gestation, and radiomic features together with deep-learning-derived image characteristics were extracted from routine 2-dimensional ultrasound scans. The extracted features characterized geometric parameters, intensity features for pixel grey levels, and grey level co-occurrence matrices, grey level size zone matrices, grey level run length matrices, neighboring grey tone difference matrices, and grey level dependence matrices, as well as filtering methods that included Laplacian of Gaussian, Wavelet, Square, Square Root, Logarithm, Exponential, Gradient, and Local Binary Pattern 2D. The authors developed machine-learning models that demonstrated good predictive performance for later GDM, particularly when maternal clinical variables were involved. The results suggest that subtle placental microstructural abnormalities are already detectable during the first trimester in pregnancies that subsequently develop GDM. These findings support the emerging role of radiomics and deep learning as tools for identifying early placental alterations that may precede the clinical onset of maternal hyperglycemia. However, the biological significance of the extracted imaging features remains incompletely understood, and prospective validation is required before widespread clinical implementation.

### 3.2. Fetus

Regarding fetal ultrasonographic findings, nine studies were included (Table 3). Fetal markers assessed included abdominal circumference (AC) and its growth velocity, head circumference (HC), biparietal diameter (BPD), femur length (FL), estimated fetal weight (EFW), fetal abdominal overgrowth ratio, anterior abdominal wall thickness (AAWT), transcerebellar diameter (TCD) and head circumference: abdominal circumference ratio (HC:AC).

#### 3.2.1. Fetal Biometry and Estimated Weight

Jin et al. [25] conducted a large retrospective cohort study including 44,179 singleton pregnancies to investigate whether routine fetal biometry performed at 22–24 weeks’ gestation could predict subsequent GDM diagnosed by a 75 g oral glucose tolerance test at 24–28 weeks. Fetal head circumference, abdominal circumference, femur length, and estimated fetal weight were measured using standardized ultrasound protocols. Of the study population, 8324 women (18.8%) subsequently developed GDM. After adjustment for maternal age, parity, fetal sex, maternal height, and pre-pregnancy body mass index (BMI_, fetuses with HC, FL, and EFW below the 10th percentile were associated with a 13–17% increased risk of subsequent maternal GDM (adjusted OR 1.15, 95% CI 1.06–1.25; adjusted OR 1.17, 95% CI 1.08–1.27; and adjusted OR 1.13, 95% CI 1.03–1.25). In contrast, abdominal circumference below the 10th percentile was not associated with GDM risk, and none of the biometric parameters above the 90th percentile predicted subsequent GDM. Moreover, the association between fetal undergrowth and later GDM appeared more pronounced among multiparous women, whereas maternal age and pre-pregnancy BMI did not significantly modify these associations. Despite these statistically significant findings, mean fetal biometric differences between pregnancies that later developed GDM and those that remained normoglycemic were minimal before 24 weeks, with less than 1 mm difference in HC, AC, and FL and less than 5 g of the EFW. The authors proposed that subtle fetal undergrowth during the mid-second trimester may precede maternal GDM diagnosis and suggested that abnormalities at the lower end of the fetal growth distribution may be more informative than average biometric measurements for identifying women at increased risk.

On the other side of the spectrum, Andersen et al. [26] conducted a retrospective cohort study of 2697 singleton pregnancies to evaluate whether fetal biometry at the routine second-trimester anatomy scan (20 + 0 to 20 + 6 weeks) was associated with subsequent GDM. GDM was diagnosed using a selective screening strategy based on a 75-g oral glucose tolerance test, with most women undergoing testing between 24 and 28 weeks’ gestation. Ultrasound measurements included head circumference, abdominal circumference, femur length, and estimated fetal weight. Overall, 174 women (6.5%) developed GDM. Fetuses with biometric measurements above the 90th percentile demonstrated a significantly increased risk of subsequent GDM, with odds ratios of 2.07 for femur length, 1.89 for head circumference, 1.63 for abdominal circumference, and 1.64 for estimated fetal weight. After adjustment for maternal obesity, the associations remained statistically significant for HC (adjusted OR 1.56, 95% CI 1.08–2.25) and FL (adjusted OR 1.57, 95% CI 1.00–2.46), whereas the associations for AC and EFW were attenuated and no longer significant. Although the mean increase in estimated fetal weight among pregnancies that later developed GDM was modest (approximately 4.2 g), the findings suggest that symmetric fetal overgrowth may already be detectable at the routine second-trimester scan before the clinical diagnosis of GDM. The authors proposed that early fetal overgrowth reflects exposure to maternal metabolic abnormalities preceding overt hyperglycemia and argued that these observations support consideration of earlier GDM screening.

Quaresima et al. [27] conducted a retrospective population-based cohort study involving 769 singleton pregnancies to investigate whether fetal growth acceleration could be detected at the routine second mid-trimester anomaly scan before the diagnosis of gestational diabetes mellitus and whether the timing of selective GDM screening recommended by the Italian National Health Service (NHS) was adequate to prevent fetal macrosomia. Women underwent fetal ultrasound at 19–21 weeks’ gestation, with biometric assessment including head circumference, biparietal diameter, abdominal circumference, femur length, and estimated fetal weight, calculated using the Hadlock formula and interpreted according to WHO fetal growth standards. GDM screening was performed with a 75-g oral glucose tolerance test at either 16–18 weeks for high-risk women or 24–28 weeks for medium- and low-risk women according to Italian National Health Service recommendations and IADPSG diagnostic criteria. Of the 769 pregnancies, 219 (28.5%) were diagnosed with GDM. At the anomaly scan, women in the medium- and low-risk groups who were subsequently diagnosed with GDM already demonstrated significantly greater AC and EFW percentiles than normoglycemic women, indicating that fetal growth acceleration had begun before routine diagnosis. High-risk women whose diagnosis was delayed until 24–28 weeks exhibited substantially higher AC and EFW than both normoglycemic women and medium-/low-risk women with GDM, indicating more pronounced fetal overgrowth when early screening recommendations were not followed. The authors concluded that although subtle fetal overgrowth is already detectable by 20 weeks in pregnancies that later develop GDM, current selective screening at 24–28 weeks appears sufficient to normalize birth weight in medium- and low-risk pregnancies, whereas earlier screening at 16–18 weeks should be reserved for high-risk women to prevent excessive fetal growth.

Liao et al. [28] conducted a retrospective case–control study involving 261 singleton pregnancies (130 women who subsequently developed GDM and 131 normoglycemic controls) to determine whether estimated fetal weight measured during the routine second-trimester ultrasound (18–22 weeks) could predict subsequent GDM or neonatal macrosomia. EFW was calculated using the Hadlock formula, and GDM was diagnosed at 24–28 weeks following a 50-g glucose challenge test with confirming 75-g oral glucose tolerance testing when indicated. After adjustment for gestational age at ultrasound, fetal sex, maternal age, ethnicity, parity, and pre-pregnancy BMI, EFW at 18–22 weeks was not associated with the subsequent development of GDM (adjusted OR 1.00, 95% CI 0.61–1.66). The authors concluded that second-trimester EFW alone is insufficient for predicting GDM, although it remains a useful predictor of fetal overgrowth at birth.

#### 3.2.2. Fetal Growth Velocity

Sovio et al. [29] conducted a large prospective cohort study of 4069 unselected nulliparous singleton pregnancies to investigate whether fetal overgrowth precedes the diagnosis of gestational diabetes mellitus. Fetal ultrasound examinations were performed at 20 and 28 weeks’ gestation, with measurement of abdominal circumference and head circumference, while GDM was diagnosed after 28 weeks according to routine clinical practice. No significant differences in fetal biometry were observed at the 20-week examination between pregnancies that subsequently developed GDM and normoglycemic controls. However, by 28 weeks, before or around the time of GDM diagnosis, fetuses of women with GDM had a twofold increased risk of AC above the 90th percentile (adjusted relative risk [aRR] 2.05, 95% CI 1.37–3.07) and a low HC:AC ratio (<10th percentile; aRR 1.97, 95% CI 1.30–2.99), indicating preferential abdominal growth. Maternal obesity exerted an independent effect of similar magnitude, and the combination of obesity and GDM resulted in an approximately fivefold increased risk of excessive fetal abdominal growth. The authors concluded that accelerated fetal abdominal growth occurs between 20 and 28 weeks, preceding the conventional diagnosis of GDM, and that maternal obesity and GDM have additive effects on fetal growth.

Wang et al. [30] conducted a prospective birth cohort study including 1023 singleton pregnancies to investigate the effect of GDM on longitudinal fetal growth trajectories in women stratified according to baseline GDM risk. Women underwent serial ultrasound examinations at 17, 24, 31, and 35 weeks’ gestation, with measurements of head circumference, abdominal circumference, biparietal diameter, femur length, and estimated fetal weight. GDM was diagnosed using a 75-g oral glucose tolerance test performed between 24 and 28 weeks according to IADPSG criteria. Rather than evaluating isolated fetal measurements, the investigators calculated fetal growth velocity between consecutive ultrasound examinations. Among both low-risk and medium-to-high-risk women, pregnancies that subsequently developed GDM demonstrated significantly accelerated abdominal circumference and estimated fetal weight growth between 17 and 24 weeks, indicating that altered fetal growth precedes the conventional timing of GDM diagnosis. The authors concluded that fetal growth acceleration begins before the routine diagnosis of GDM and is more pronounced and sustained among women with established metabolic risk factors, supporting the concept of risk-stratified early GDM screening.

#### 3.2.3. Fetal Abdomen

Kim et al. [31] conducted a large retrospective cohort study including 6996 singleton pregnancies (357 women who subsequently developed GDM and 6639 with normal glucose tolerance) to determine whether fetal abdominal overgrowth (FAO) is already present before the conventional diagnosis of GDM. Routine fetal biometry was performed at 20–24 weeks’ gestation, approximately four weeks before universal GDM screening at 24–28 weeks using a 50-g glucose challenge test followed by a diagnostic 100-g oral glucose tolerance test. Rather than relying solely on conventional fetal biometric parameters, the investigators calculated fetal abdominal overgrowth ratios (FAORs), which compare abdominal circumference-derived gestational age with gestational age based on the last menstrual period, biparietal diameter, or femur length, thereby assessing disproportionate abdominal growth. Overall, pregnancies that subsequently developed GDM demonstrated significantly greater abdominal growth in comparison to head and femur size, with higher FAORs than normoglycemic controls, despite similar biparietal diameter, femur length, and estimated fetal weight. Importantly, these abnormalities were confined to women who were ≥35 years of age and/or obese (BMI ≥ 25 kg/m^2^), whereas no significant differences were observed among younger, non-obese women. Fetal abdominal overgrowth became detectable from approximately 21 weeks’ gestation and persisted until GDM diagnosis. The authors concluded that fetal abdominal adiposity develops before the biochemical diagnosis of GDM in older and/or obese women, suggesting that the current screening strategy at 24–28 weeks may be too late to prevent early fetal overgrowth in high-risk pregnancies.

Venkataraman et al. [15] conducted a retrospective case–control study including 331 singleton pregnancies (153 women who subsequently developed GDM) with the objective to investigate whether fetal adiposity is detectable before the biochemical diagnosis of GDM. Fetal ultrasound examinations were performed at 12, 20, and 32 weeks’ gestation, with assessment of conventional biometric parameters, including head circumference, abdominal circumference, femur length, and biparietal diameter, together with anterior abdominal wall thickness (AAWT), a validated surrogate marker of fetal abdominal adiposity. GDM was diagnosed between 24 and 28 weeks using a 75-g oral glucose tolerance test according to IADPSG criteria. At 12 weeks, no differences in fetal biometry were observed between groups. However, at approximately 20 weeks, before routine GDM diagnosis, fetuses of women who subsequently developed GDM demonstrated significantly greater AAWT despite significantly smaller HC, AC, FL, and BPD after adjustment for maternal age, BMI, parity, gestational weight gain, fetal sex, and gestational age. The authors proposed that these findings represent an early fetal “thin–fat” phenotype, characterized by preferential adipose tissue deposition despite relatively smaller lean body mass, suggesting that fetal body composition may be altered several weeks before the conventional diagnosis of GDM.

#### 3.2.4. Nuchal Translucency

Although first-trimester nuchal translucency (NT) has been evaluated as a potential early sonographic marker of GDM, a prospective study [32] of 464 singleton pregnancies found no significant difference in NT thickness at 11–14 weeks between women who subsequently developed GDM and those with normal glucose tolerance after adjustment for crown-rump length. Multivariable analysis confirmed that NT was related only to crown-rump length (CRL) and not to maternal GDM status, BMI, or age. These findings suggest that first-trimester NT does not reflect the early fetal adaptations associated with maternal dysglycemia and is unlikely to have clinical utility as a screening marker for subsequent GDM.

### 3.3. Uterine Artery Pulsatility Index

When searching for relevant uterine artery early sonographic markers, pulsatility index was linked to gestational diabetes mellitus in seven studies (Table 4).

One of the earliest studies exploring uterine artery Doppler and gestational diabetes was the prospective cohort study by van den Elzen et al. [33], which followed 352 pregnant women aged ≥35 years and measured uterine artery pulsatility indices at 7–11, 12–13, and 23–27 weeks’ gestation. Women with first-trimester uterine artery PI values in the highest quartile at 12–13 weeks had an approximately ninefold greater risk of subsequently developing GDM than those in the lowest quartile (RR 8.7, 95% CI 1.1–71.3). However, only 17 cases of GDM occurred, the study population consisted exclusively of women of advanced maternal age, and diabetes was evaluated as one of several obstetric outcomes rather than the primary endpoint. Consequently, although these findings suggested a possible association between increased first-trimester uterine artery resistance and later GDM, the results have not been consistently reproduced in more recent studies using contemporary diagnostic criteria.

Chatzakis et al. [34] conducted a retrospective case–control study to investigate whether first-trimester uterine artery Doppler differs in pregnancies complicated by gestational diabetes mellitus, type 1 diabetes mellitus (T1DM), type 2 diabetes mellitus (T2DM), and uncomplicated pregnancies. From an initial cohort of 15,638 pregnancies, 65 women with GDM, 58 with T1DM, 67 with T2DM, and 65 healthy controls were included. All participants underwent first-trimester ultrasound screening, during which uterine artery pulsatility index (UtA PI) was measured by Fetal Medicine Foundation-certified sonographers and expressed as multiples of median (MoM). GDM was subsequently diagnosed between 24 and 28 weeks according to IADPSG criteria. Mean UtA PI values were similar across all groups (1.02 ± 0.31 MoM in GDM, 1.00 ± 0.26 MoM in T1DM, 1.04 ± 0.32 MoM in T2DM, and 1.08 ± 0.33 MoM in controls), with no statistically significant differences. Multivariable linear regression adjusting for maternal age, BMI, smoking status, parity, and method of conception likewise demonstrated no independent association between diabetes status and first-trimester UtA PI. The authors concluded that, despite the recognized placental dysfunction associated with diabetes, first-trimester uterine artery resistance does not appear to be altered in pregnancies that subsequently develop GDM or in women with pre-existing diabetes, suggesting that UtA Doppler is unlikely to be a useful early screening marker for diabetic pregnancies.

Tenenbaum-Gavish et al. [35] conducted a prospective cohort study within the Combined Multimarker Screening and Randomized Patient Treatment with Aspirin for Evidence-Based Preeclampsia Prevention (ASPRE) trial to evaluate whether first-trimester clinical, placental, inflammatory, and biophysical markers could predict the subsequent development of gestational diabetes mellitus. The study included 205 singleton pregnancies, comprising 20 women who later developed GDM and 185 uncomplicated controls. At 11 + 0 to 13 + 6 weeks’ gestation, participants underwent standardized first-trimester screening including measurement of mean uterine artery pulsatility index, mean arterial pressure, and maternal serum biomarkers. Although raw UtA-PI values did not differ significantly between groups, gestational age-adjusted UtA-PI expressed as multiples of the median (MoM) was significantly higher in women who subsequently developed GDM (1.13 vs. 1.00 MoM, *p* = 0.015), suggesting mildly increased uteroplacental vascular resistance early in pregnancies destined to develop GDM. However, UtA-PI alone demonstrated limited predictive performance (AUC 0.63), with a detection rate of only 28% at a 10% false-positive rate. The authors concluded that while first-trimester UtA-PI may reflect subtle alterations in placental perfusion in pregnancies that later develop GDM, its value as an isolated screening marker is limited.

Sweeting et al. [36] conducted a large case–control study including 980 singleton pregnancies (248 women with GDM and 732 controls) to investigate whether routinely collected first-trimester aneuploidy and pre-eclampsia screening markers could improve early prediction of gestational diabetes mellitus. Women underwent standardized first-trimester assessment between 11 and 13 + 6 weeks’ gestation, including measurement of uterine artery pulsatility index, mean arterial pressure, pregnancy-associated plasma protein A (PAPP-A), and free β-human chorionic gonadotropin (free β-hCG). For women diagnosed with standard GDM (≥24 weeks’ gestation), first-trimester UtA-PI MoM was not significantly different from controls (1.00 [IQR 0.83–1.18] vs. 1.05 [IQR 0.84–1.29]), indicating that uterine artery Doppler abnormalities were not evident before diagnosis in this subgroup. Although lower UtA-PI was identified as an independent predictor in the overall multivariable model that combined early- and standard-onset GDM, the absence of a significant association in women diagnosed after 24 weeks suggests that first-trimester uterine artery Doppler contributes little to the prediction of conventional GDM. The addition of first-trimester biomarkers to maternal clinical risk factors increased the predictive model AUC only modestly, from 0.88 to 0.90, further supporting the limited incremental value of UtA-PI as an isolated screening marker.

Tranidou et al. [37] conducted a large prospective cohort study involving 4917 singleton pregnancies to develop first-trimester prediction models for gestational diabetes mellitus using maternal characteristics, obstetric history, biochemical markers, and uterine artery Doppler findings. Women underwent routine first-trimester screening between 11 + 0 and 13 + 6 weeks’ gestation. Universal GDM screening was subsequently performed at 24–28 weeks using a 75-g oral glucose tolerance test based on HAPO diagnostic criteria. Women who later developed GDM had significantly lower first-trimester UtA-PI z-scores than normoglycemic controls (*p* = 0.03). In multivariable analysis including maternal characteristics and pregnancy biomarkers, lower UtA-PI remained independently associated with subsequent GDM (adjusted OR 0.89, 95% CI 0.80–0.98; *p* = 0.02). However, after incorporation of maternal demographic, medical, and obstetric risk factors into the comprehensive prediction model, UtA-PI was no longer an independent predictor (adjusted OR 0.94, 95% CI 0.85–1.04; *p* = 0.22). Furthermore, the model based on biomarkers and UtA-PI demonstrated the lowest predictive performance of all evaluated models, whereas models relying primarily on maternal clinical characteristics achieved superior discrimination. The authors concluded that although first-trimester uterine artery Doppler may reflect subtle placental alterations associated with pregnancies that later develop GDM, its incremental contribution to early risk prediction is limited.

Shen et al. [38] performed a secondary analysis of a large prospective cohort including 9372 singleton pregnancies undergoing routine first-trimester screening between 11 and 13 + 6 weeks’ gestation to evaluate whether maternal characteristics, obstetric history, and preeclampsia screening biomarkers could predict subsequent gestational diabetes mellitus. GDM was diagnosed later in pregnancy using a 75-g oral glucose tolerance test according to WHO 2013 criteria in women identified as being at increased clinical risk. First-trimester assessment included mean arterial pressure, uterine artery pulsatility index, pregnancy-associated plasma protein-A, and placental growth factor, standardized as MoM. Among the 9372 pregnancies, 837 (8.9%) subsequently developed GDM. While women who later developed GDM demonstrated significantly higher mean arterial pressure and lower PAPP-A levels, UtA-PI did not differ significantly between women with and without GDM (log10 UtA-PI MoM, *p* = 0.451). Accordingly, UtA-PI was not retained as an independent predictor in multivariable regression modelling. The authors concluded that uterine artery Doppler does not contribute meaningful incremental predictive value.

Wong et al. [18] conducted a prospective case–control study of 155 singleton pregnancies at increased risk for gestational diabetes mellitus, including 31 women who subsequently developed GDM diagnosed by universal screening at 24–28 weeks. Bilateral uterine artery pulsatility index was measured during both the first (11–13 + 6 weeks) and second (21–23 + 6 weeks) trimesters. No significant differences in uterine artery PI were observed between women who later developed GDM and normoglycemic controls in either trimester (first trimester: right PI *p* = 0.257, left PI *p* = 0.677; second trimester: right PI *p* = 0.599, left PI *p* = 0.213). Uterine artery PI was not associated with subsequent GDM on multivariable analysis and was not identified as an independent predictor of disease. The authors concluded that uterine artery Doppler does not appear to reflect the early placental alterations associated with GDM, supporting its limited utility as a standalone screening marker for pregnancies that subsequently develop GDM.

## 4. Discussion

Although current guidelines recommend universal screening between 24 and 28 weeks of gestation, there is increasing recognition that the pathophysiological processes underlying GDM begin much earlier in pregnancy, before hyperglycemia reaches diagnostic thresholds [39,40]. Normal pregnancy is characterized by dynamic metabolic adaptations that ensure an adequate nutrient supply for fetal growth. During early pregnancy, maternal insulin sensitivity is maintained or might increase slightly, promoting maternal energy storage and adipose tissue accumulation. As gestation progresses, placental hormones, including human placental lactogen, placental growth hormone, progesterone, estrogen, prolactin, cortisol, and inflammatory cytokines, induce progressive insulin resistance, which may reduce insulin sensitivity during late pregnancy [1,41,42,43]. To maintain euglycemia, pancreatic β-cells undergo adaptive changes, including increased insulin secretion and, potentially, β-cell hyperplasia and hypertrophy [41]. In women with underlying β-cell dysfunction or pre-existing insulin resistance, such as those with obesity or other metabolic risk factors, these compensatory mechanisms become insufficient. Consequently, maternal insulin secretion does not meet the increasing metabolic demands of pregnancy, resulting in hyperglycemia and the development of gestational diabetes mellitus [44].

The observed sonographic abnormalities may reflect early placental and fetal adaptations to the metabolic environment preceding the clinical diagnosis of GDM. Maternal insulin resistance, which progressively increases during pregnancy, may be accompanied by alterations in placental trophoblast function, angiogenesis, and nutrient transport before maternal hyperglycemia becomes clinically apparent [45]. Reduced placental vascularization observed by three-dimensional power Doppler might reflect alterations in placental microvascular development or remodeling, potentially affecting the exchange between the fetus and the mother. Moreover, increased placental stiffness detected by elastography may indicate changes in tissue composition or extracellular matrix properties, although the specific histopathological correlates of increased strain ratio are still to be established. The abnormal placental texture identified by radiomic and artificial intelligence-based approaches may capture complex differences in tissue organization that are not appreciable by conventional ultrasound; however, their biological interpretation remains uncertain.

The fetal findings may be explained, at least partially, by altered nutrient availability and fetal metabolic adaptation. Increased maternal glucose and other nutrient availability may stimulate fetal insulin secretion, promoting preferential deposition of adipose tissue, particularly in the abdominal compartment [46,47]. This mechanism is consistent with the increased anterior abdominal wall thickness, abdominal overgrowth indices, and accelerated abdominal circumference observed before conventional GDM diagnosis. Importantly, studies evaluating conventional fetal biometry have reported both smaller and larger fetal measurements [7]. These apparently discrepant findings may reflect differences in gestational age, maternal metabolic phenotype, obesity, population characteristics, and the specific parameters used to characterize fetal growth. Therefore, early fetal metabolic programming may not initially manifest as generalized fetal overgrowth but rather as changes in body composition or growth trajectories that precede more apparent abnormalities in fetal size.

The placenta is one of the earliest organs affected by this maternal metabolic dysfunction, undergoing structural and functional adaptations in response to hyperglycemia [48]. However, the majority of ultrasound studies have evaluated placental changes after the diagnosis of GDM, with relatively few investigating whether these abnormalities are detectable before the conventional diagnostic window. Multiple studies [49,50,51,52,53,54] demonstrated that pregnancies complicated by GDM had significantly larger placental volumes and higher estimated fetal weight-to-placental volume ratios during mid-pregnancy compared with healthy controls, as well as reduced placental vascular indices or immature placental appearance. Because the participants already had an established diagnosis of GDM, the applicability of these findings as predictive markers remained limited.

Di Filippo et al. [55] recently performed a comprehensive systematic review of 174 studies evaluating diagnostic biomarkers for GDM, including 27 studies investigating ultrasound features, highlighting the growing interest in imaging-based approaches despite their relative underrepresentation compared with biochemical markers. Importantly, the review primarily evaluated ultrasound findings obtained after the second trimester and in women with established GDM, whereas the predictive value of sonographic markers identified before the conventional diagnostic window received insufficient attention. The ultrasound biomarkers identified maternal, fetal, placental, and combined parameters. Frequently reported findings included increased maternal visceral and subcutaneous adipose tissue, fetal abdominal wall and subcutaneous fat thickness, fetal liver volume, fetal epicardial fat thickness, placental thickness, and alterations in umbilical vein and fetal Doppler parameters. Among the ultrasound markers assessed directly against the OGTT, fetal subcutaneous adipose tissue thickness demonstrated the most promising diagnostic performance. Among the ultrasound markers assessed directly against the OGTT, fetal subcutaneous adipose tissue thickness and the UGDS score (increased adipose subcutaneous tissue, cardiac width, cardiac circumference, placental thickness, polyhydramnios, asymmetrical macrosomia, thickened intra-ventricular septum, intensified breathing movements, immature appearance of placenta) demonstrated the most promising diagnostic performance. In another study, women with abnormal glucose tolerance tests exhibited significantly increased fetal subcutaneous fat thickness at multiple anatomical sites compared with controls, indicating that maternal dysglycemia is associated with altered fetal body composition [56]. Sabir et al. [57] evaluated fetal anterior abdominal wall thickness in women at increased risk of GDM and found that it accurately identified GDM, with a sensitivity of 93.1% and specificity of 82.7%. Much like the previously mentioned studies, as ultrasound assessment and GDM diagnosis were performed concurrently, the studies could not determine whether increased AAWT precedes or results from maternal hyperglycemia.

Therefore, by the time the standard oral glucose tolerance test is performed, maternal metabolic derangements may have already influenced placental development and fetal growth. GDM diagnosed before 26 weeks has been associated with a higher risk of fetal overgrowth, including accelerated abdominal circumference and increased birth weight, as well as higher offspring BMI during early childhood [58]. These findings likely reflect prolonged fetal exposure to maternal hyperglycemia and a greater inflammatory burden, particularly when early GDM coexists with excessive gestational weight gain. This data supports that fetal metabolic alterations begin before the conventional 24–28-week screening period, underscoring the need for earlier identification of pregnancies at risk [59]. Pregnancy complications are associated not only with adverse maternal and fetal health outcomes, but also with substantial psychological distress. Receiving a diagnosis during pregnancy often evokes feelings such as anxiety, fear, uncertainty, and concerns about the baby’s health, which may negatively affect maternal well-being and family functioning [60]. Prenatal examinations are an opportunity to detect subclinical maternal diseases, and that asymptomatic maternal conditions can lead to severe neonatal complications if left unrecognized [61]. Early identification of pregnancies at risk for GDM is essential, as it provides an opportunity for timely intervention before maternal hyperglycaemia results in irreversible placental and fetal adaptations.

The available evidence discussed in this review consistently suggests that placental structural and vascular alterations precede the clinical diagnosis of GDM and are detectable from the late first trimester. Across multiple prospective studies, pregnancies that subsequently developed GDM exhibited reduced placental vascularization, reflected by significantly lower three-dimensional power Doppler vascular indices, particularly the vascularization–flow index, compared with normoglycemic pregnancies. Among the Doppler-derived parameters, VFI emerged as the most reproducible and robust marker, remaining independently associated with subsequent GDM after adjustment for maternal confounders in several studies. In contrast, flow index demonstrated inconsistent associations, suggesting that early GDM may primarily affect placental vascular architecture rather than blood flow intensity. Similarly, placental volume showed variable findings, with some studies reporting reduced first-trimester placental volume, whereas others observed enlargement only during the second trimester, indicating that volumetric changes may represent a later manifestation of placental adaptation than alterations in vascularization.

Beyond vascularization, emerging evidence indicates that placental morphology and biomechanical properties may also be altered early in pregnancies complicated by GDM. Increased placental thickness during the early second trimester and elevated placental strain ratio measured by sonoelastography in the first trimester were both independently associated with subsequent GDM, supporting the hypothesis that structural remodeling and altered tissue elasticity occur before overt maternal hyperglycemia. These findings reinforce the concept that placental dysfunction is an early event in the pathophysiology of GDM and that advanced ultrasound techniques may detect subtle microstructural and functional changes before conventional biochemical diagnosis.

Evidence regarding placental biomechanical properties is currently limited but suggests that alterations in placental tissue characteristics may occur even earlier than structural or vascular changes. In the only prospective study identified, increased first-trimester placental strain ratio measured by real-time sonoelastography independently predicted subsequent GDM, indicating that increased placental stiffness may represent an early manifestation of placental dysfunction preceding the onset of maternal hyperglycemia.

Recent advances in artificial intelligence have further expanded the evaluation of early placental abnormalities in pregnancies destined to develop GDM. Two multicenter retrospective studies demonstrated that quantitative analysis of routine first-trimester placental ultrasound images using radiomics and deep-learning algorithms can identify subtle placental texture and microstructural alterations before the clinical diagnosis of GDM. In both studies, predictive performance improved when placental imaging features were combined with maternal clinical characteristics, achieving good discrimination in both internal and external validation cohorts. These findings suggest that placental microarchitectural changes, which are not appreciable by conventional ultrasound assessment, may represent some of the earliest imaging manifestations of GDM.

The evidence regarding conventional fetal biometry before the diagnosis of GDM is inconsistent. Several studies reported that pregnancies subsequently complicated by GDM already exhibit subtle alterations in fetal growth during the mid-second trimester, although the pattern of these changes varies considerably. Andersen et al. [26] and Quaresima et al. [27] observed increased fetal biometry, particularly abdominal circumference and estimated fetal weight, before routine GDM diagnosis, suggesting that accelerated fetal growth may begin before overt maternal hyperglycemia is recognized. In contrast, Jin et al. [25], in the largest cohort to date, found that fetuses with smaller head circumference, femur length, and EFW were at a modestly increased risk of subsequent GDM, while AC was not associated with disease risk. As a result, this study complements later longitudinal studies showing fetal overgrowth after GDM diagnosis and supports the concept that fetal growth trajectories, rather than isolated biometric measurements, may better reflect the evolving pathophysiology of GDM. Liao et al. [28] further demonstrated that EFW alone at 18–22 weeks was not predictive of later GDM despite being strongly associated with neonatal macrosomia.

Collectively, these findings indicate that although subtle alterations in fetal growth may precede the diagnosis of GDM, routine fetal biometry alone has limited predictive value. More specific markers of fetal body composition or growth patterns, particularly those reflecting abdominal adiposity rather than overall fetal size, may provide greater clinical utility for early detection.

On that note, evidence regarding fetal growth velocity is more consistent than that for isolated fetal biometric measurements. Both Sovio et al. [29] and Wang et al. [30] demonstrated that accelerated fetal abdominal growth precedes the conventional diagnosis of GDM, supporting the concept that fetal growth abnormalities emerge before maternal hyperglycemia is clinically recognized. Sovio et al. [29] showed that while fetal biometry was comparable at 20 weeks, fetuses of women who later developed GDM exhibited preferential abdominal growth and a reduced HC:AC ratio by 28 weeks, indicating disproportionate fetal growth. Similarly, Wang [30] found significantly increased abdominal circumference and estimated fetal weight growth velocity between 17 and 24 weeks, even before routine GDM screening, with more sustained acceleration among women at higher metabolic risk. Dynamic assessment of fetal growth may be more informative than single biometric measurements for identifying pregnancies destined to develop GDM. Both studies also highlight the central role of fetal abdominal growth as the earliest manifestation of altered fetal development, supporting the hypothesis that maternal metabolic disturbances influence fetal growth several weeks before GDM is clinically diagnosed. Kim et al. [31] and Venkataraman et al. [15] independently demonstrated that abnormalities in fetal abdominal fat deposition are detectable around 20–24 weeks, before routine biochemical diagnosis. Kim reported disproportionate fetal abdominal overgrowth despite otherwise normal fetal size, particularly among older and obese women, whereas Venkataraman observed increased anterior abdominal wall thickness despite smaller conventional biometric measurements, describing an early “thin–fat” fetal phenotype. Although these markers appear promising, both studies were retrospective and conducted in specific ethnic populations, highlighting the need for prospective validation in more diverse cohorts before they can be incorporated into routine clinical practice.

In contrast, the available evidence does not support first-trimester nuchal translucency as an early sonographic marker of GDM. The only study evaluating this parameter found no association between NT thickness and subsequent GDM after adjustment for crown-rump length, suggesting that early fetal adaptations to maternal dysglycemia are reflected by changes in body composition rather than by first-trimester markers routinely used for aneuploidy screening. Consequently, NT appears to have limited value for early GDM prediction.

Despite these encouraging findings, the available evidence should be interpreted with caution. Most studies were single-center investigations with relatively small sample sizes and were conducted in women already considered at increased risk for GDM, limiting generalizability to unselected obstetric populations. Furthermore, ultrasound methodologies varied considerably, including differences in equipment, acquisition protocols, and analyzed parameters, which may explain some of the heterogeneity in reported results. Most predictive models also lacked external validation, preventing firm conclusions regarding their clinical applicability. Consequently, while some of these markers appear promising for early GDM risk stratification, larger multicenter prospective studies using standardized imaging protocols are required before these techniques can be incorporated into routine prenatal screening.

While outside the scope of fetal biometric assessment, an observational study [62] of 603 singleton pregnancies reported that an increased first-trimester fetal heart rate (FHR) at 11–14 weeks was independently associated with the subsequent development of GDM. First-trimester FHR demonstrated good predictive performance (AUC 0.809), and an FHR threshold of 162 beats/min yielded a detection rate of 76.9% with a negative predictive value of 85.5% for later GDM. These findings suggest that functional fetal adaptations to maternal metabolic disturbances may be detectable before conventional GDM diagnosis. Consequently, FHR should be regarded as an exploratory functional marker.

The evidence supporting uterine artery pulsatility index as an early marker of GDM is limited and largely inconsistent. While an early study by van den Elzen et al. [33] suggested that increased first-trimester UtA-PI was associated with a higher risk of subsequent GDM, this finding was based on a small number of cases in an older, high-risk population and has not been replicated in contemporary cohorts. Most subsequent studies, including large prospective and retrospective cohorts, reported no significant differences in first-trimester UtA-PI between pregnancies that later developed GDM and normoglycemic controls, particularly among women with conventional GDM diagnosed after 24 weeks’ gestation. Overall, the current evidence suggests that uterine artery Doppler has limited value as an independent screening tool for GDM. Across studies, the addition of UtA-PI to prediction models resulted in minimal or no improvement beyond established maternal risk factors and biochemical markers.

From a translational perspective, while fetal biometry and placental thickness can be readily incorporated into routine ultrasound examinations, three-dimensional power Doppler and placental elastography require specialized equipment, standardized acquisition protocols, and operator expertise. Radiomic and deep-learning approaches impose additional requirements for image processing, computational infrastructure, and reproducible analytical pipelines. Differences in equipment, acquisition settings, operator experience, and post-processing may affect reproducibility, while validated reference ranges and clinically applicable thresholds remain unavailable for several emerging markers. Moreover, the cost-effectiveness of these approaches has not yet been established. Their current value is primarily investigational.

Our narrative review has several limitations. First, the available evidence is heterogeneous with respect to study design, study populations, diagnostic criteria for GDM, ultrasound protocols, and the sonographic parameters evaluated, limiting direct comparison between studies. This heterogeneity also includes differences in the gestational age at ultrasound assessment, maternal BMI and baseline metabolic risk, ethnicity, GDM screening strategies, adjustment for potential confounders, and timing of GDM diagnosis and therapeutic intervention, which may contribute to the apparently divergent findings regarding early fetal undergrowth versus accelerated fetal growth. Diagnostic approaches varied from selective to universal screening and employed different criteria (IADPSG, WHO, Carpenter–Coustan, and national guidelines), which may have influenced the reported associations. Secondly, many studies were retrospective, single-center investigations with relatively small sample sizes and limited ethnic diversity, reducing the generalizability of their findings. Differences in maternal metabolic characteristics and population risk profiles may also modify the relationship between early ultrasound findings and subsequent GDM, while treatment initiated after diagnosis may influence fetal growth trajectories observed in studies performed later in pregnancy. Thirdly, substantial methodological heterogeneity existed in ultrasound acquisition and analysis, particularly for advanced techniques such as three-dimensional power Doppler, elastography, and artificial intelligence-based radiomics, where standardized acquisition protocols and validated diagnostic thresholds are lacking. Finally, because this is a narrative review, formal assessment of methodological quality, risk of bias, publication bias, and quantitative meta-analysis were not performed, and the conclusions should be interpreted as a qualitative synthesis of the current evidence.

Despite these limitations, the review provides a comprehensive overview of the current evidence regarding early placental and fetal sonographic markers of GDM and highlights areas requiring validation in large, prospective, multicenter studies using standardized imaging and diagnostic protocols.

Future research should prioritize large, prospective, multicenter studies with standardized ultrasound acquisition protocols and uniform GDM diagnostic criteria to improve the comparability and reproducibility of findings. They should determine whether incorporating early sonographic markers into existing clinical prediction models improves risk stratification, enables earlier intervention, and ultimately translates into better maternal and neonatal outcomes.

## 5. Conclusions

Current evidence suggests that sonographic placental and fetal alterations are detectable before the conventional diagnosis of gestational diabetes mellitus. Among placental markers, three-dimensional power Doppler indices, particularly the vascularization–flow index, consistently demonstrated reduced placental vascularization in pregnancies that later developed GDM, while placental elastography and artificial intelligence-based radiomic analysis represent promising emerging techniques. In contrast, uterine artery Doppler has shown limited and inconsistent predictive value, suggesting that early placental dysfunction is more closely related to microvascular and structural changes than to altered uteroplacental perfusion. Fetal findings indicate that conventional biometry alone has limited predictive utility, whereas longitudinal assessment of fetal growth and markers of fetal adiposity, including anterior abdominal wall thickness and abdominal overgrowth indices, more consistently identify early fetal adaptations to maternal dysglycemia. However, current evidence is limited by methodological heterogeneity, predominantly retrospective study designs, and a lack of external validation. Large prospective multicenter studies using standardized imaging protocols are needed to determine the clinical utility of these sonographic markers and their potential role in early GDM screening and risk stratification.

## Figures and Tables

**Table 1:** Search strategy for each of the outcomes, as well as number of articles found from each database.

	PubMed	SCOPUS	Medline
Gestational diabetes mellitus and placental findings	305	506	152
Gestational diabetes mellitus and uterine artery findings	43	88	50
Gestational diabetes mellitus and fetal findings	460	238	129
Total	808	832	331
	1971		

**Table 2:** Studies discussing early ultrasonographic placental characteristics in the context of gestational diabetes mellitus.

Study	Design/Population	Gestational Age (Weeks + Days)	Placental Marker Assessed	Results	Limitations
Wong 2019 [18]	Prospective case–control, 155	11 + 0 to 23 + 6	VI; FI; VFI; PV	31 participants developed GDM. Lower placental VI and VFI in the first and second trimesters, and increased second-trimester placental volume, were associated with GDM. First- and second-trimester VFI and second-trimester placental volume remained independent predictors after adjustment, while FI and uterine artery PI showed no significant association.	Single-center study. Small sample size.
Han 2021 [19]	Prospective, 141	11 + 0 to 13 + 6	VI; FI; VFI,	Women who developed GDM had significantly lower first-trimester values. VFI remained an independent predictor of GDM, and a combined model including maternal age, gravida, placental volume, and VFI improved predictive performance.	Single-center study with a relatively small sample size; placental assessment was limited to the first trimester, requiring external validation in larger prospective cohorts.
Zhu 2025 [20]	Retrospective, 122	11 + 0 to 13 + 6	PV; VI; FI; VFI	Women who developed GDM had significantly lower first-trimester PV, VI, FI, and VFI. Moreover, PV, VI, and FI were independent predictors of GDM, and a combined clinical + 30° ultrasound model showed the best predictive performance	Retrospective single-center study with a relatively small sample; potential selection and information bias
Lavanya 2025 [21]	Prospective cross-sectional, 139	16 + 0 to 19 + 6	Placental thickness	Placental thickness was significantly greater in women with GDM and positively correlated with maternal glycemia. Placental thickness ≥ 2.7 cm was identified as a significant predictor of GDM.	Cross-sectional design with a single placental measurement, small sample size, single observer, and potential observer/instrumental bias.
Şengül 2023 [22]	Prospective cohort, 210	11 + 0 to 14 + 0	Placental strain ratio (real-time sonoelastography)	Higher first-trimester placental strain ratio was independently associated with subsequent GDM and predicted GDM using a cut-off >0.986.	Single-center study; placental assessment limited to the first trimester; findings require validation in larger, multicenter cohorts.
Peng 2026 [23]	Retrospective multicenter, 628	11 + 0 to 13 + 6	Placental ultrasound texture (radiomic features), deep learning-derived placental features, and a combined radiomics–deep learning model.	A multimodal model incorporating placental texture features significantly improved early GDM prediction (AUC 0.879 internally; 0.817 in validation), outperforming clinical, radiomics-only, and deep learning-only models. Placental texture abnormalities in the first trimester were predictive of subsequent GDM.	Retrospective design; radiomics analysis relied on manual placental segmentation; external validation was limited to one additional center, requiring broader prospective multicenter validation before clinical implementation.
Zhou 2024 [24]	Retrospective, 415	11 + 0 to 13 + 6	Placental ultrasound radiomic texture features and deep learning (DL) features derived from 2D ultrasound images	Abnormal first-trimester placental texture features were associated with subsequent GDM. The radiomics model showed good predictive performance (AUC 0.91 discovery, 0.86 validation).	Retrospective study with a relatively small sample size; limited placental heterogeneity captured by the dataset; additional large, multicenter studies and multimodal placental assessment are needed for validation.

Abbreviations: AUC = area under curve: DL = deep learning; GDM = gestational diabetes mellitus; VI = vascularization index; FI = flow index; VFI = vascularization flow index; PV = placental volume.

**Table 3:** Studies discussing early ultrasonographic fetal characteristics in the context of gestational diabetes mellitus.

Study	Design/Population	Gestational Age (Weeks + Days)	Fetal Marker Assessed	Results	Limitations
Jin 2020 [25]	Retrospective, 44,179	22 + 0 to 24 + 0	HC, AC, FL, EFW	Fetuses with HC, FL, or EFW < 10th percentile at 22–24 weeks had a modestly increased risk of subsequent GDM, whereas fetal overgrowth (>90th percentile) was not associated with GDM.	Retrospective single-center study; pre-existing diabetes could not be excluded; findings may not be generalizable beyond the study population
Andersen 2022 [26]	Retrospective, 2697	20 + 0 to 20 + 6	HC, AC, FL, EFW	Fetal biometry > 90th percentile, particularly HC and FL, was associated with an increased risk of subsequent GDM. Findings suggest symmetrical fetal overgrowth is already present at the routine second-trimester scan before GDM diagnosis.	Retrospective design; selective GDM screening may have missed undiagnosed cases; Danish diagnostic criteria likely captured more severe GDM, limiting generalizability
Quaresima 2020 [27]	Retrospective, 219	19 + 0 to 21 + 0	HC, BPD, TCD, AC, FL, EFW	Medium- and low-risk women who later developed GDM had significantly higher AC and EFW percentiles at the anomaly scan, indicating mild early fetal growth acceleration before GDM diagnosis.	Retrospective single-center study; unequal representation of risk groups (particularly few low-risk women); findings may have limited generalizability.
Liao 2014 [28]	Case–control, 261	18 + 0 to 22 + 0	EFW	Second-trimester EFW was not associated with subsequent GDM.	Case–control design; GDM treatment may have attenuated fetal growth; EFW was estimated by ultrasound, which has inherent measurement limitations.
Sovio 2016 [29]	Prospective cohort, 4069	20, 28	AC, HC, HC:AC ratio, AC growth velocity	No differences in fetal biometry were observed at 20 weeks. By 28 weeks, pregnancies that developed GDM had increased AC, accelerated AC growth velocity, and a lower HC:AC ratio, indicating fetal overgrowth preceding GDM diagnosis.	Cohort consisted primarily of White nulliparous women, limiting generalizability; GDM diagnostic criteria changed during the study period; use of a 50-g glucose challenge test may have resulted in some undiagnosed GDM cases.
Wang 2024 [30]	Prospective cohort, 1023	17, 24, 31, 35	EFW, AC, HC, BPD, FL, fetal growth velocity, HC:AC	GDM was associated with accelerated fetal growth (particularly AC and EFW) from 17–24 weeks in both risk groups.	Pre-pregnancy BMI was self-reported; limited number and spacing of ultrasound examinations prevented precise determination of when abnormal fetal growth began.
Kim 2021 [31]	Retrospective observational, 6996	20 + 0 to 24 + 0	Fetal abdominal overgrowth ratio, AC, EFW	Fetal abdominal overgrowth was already present at 20–24 weeks, before biochemical diagnosis of GDM, in older (≥35 years) and/or obese (BMI ≥ 25 kg/m^2^) women, but not in younger, non-obese women. At 20–24 weeks, it was associated with markedly higher odds of persistent FAO at GDM diagnosis (OR 10.15) and LGA at birth (OR 5.57). The authors concluded that earlier GDM detection and intervention may be needed for high-risk women.	Single-center, retrospective observational study; all participants were Asian; inter-observer variability in ultrasound measurements was not assessed; unequal numbers of scans across gestational weeks.
Venkataraman 2017 [15]	Retrospective case–control, 331	12, 20, 32	AAWT, AC, HC, FL, BPD	Fetuses of women who later developed GDM had increased AAWT (fetal adiposity) as early as 20 weeks, despite smaller HC, AC, FL, and BPD, demonstrating a “thin–fat” phenotype before biochemical diagnosis. Increased AAWT persisted at 32 weeks and was independently associated with maternal fasting glucose	Retrospective single-center study restricted to a South Asian population; detailed data on GDM treatment and glycemic control were unavailable; glucose levels were not measured at the 20-week scan; dietary information was lacking.
Leipold 2005 [32]	Prospective cohort, 464	11 + 0 to 14 + 0	Fetal nuchal translucency	No significant association was found between first-trimester NT thickness and subsequent GDM after adjustment for crown–rump length, maternal age, and BMI.	Single-center study; women with pre-existing diabetes and fetal chromosomal abnormalities were excluded; only NT was evaluated, limiting assessment of other early fetal markers.

AAWT = anterior abdominal wall thickness; HC = head circumference; AC = abdominal circumference; FL = femur length; EFW = estimated fetal weight; BPD = biparietal diameter; TCD = transcerebellar diameter.

**Table 4:** Studies discussing early uterine artery pulsatility index in the context of gestational diabetes mellitus.

Study	Design/Population	Gestational Age (Weeks + Days)	Results	Limitations
van der Elzen 1995 [33]	Prospective cohort, 352	7–11, 12–13, and 23–27	Increased uterine artery PI at 12–13 weeks was associated with a higher risk of subsequent GDM, with women in the highest PI quartile having an approximately 8-fold increased risk compared with those in the lowest quartile.	Study included only women aged ≥35 years, limiting generalizability; relatively few GDM cases; Doppler PI alone showed limited predictive performance and was not recommended as a standalone screening test.
Chatzakis 2023 [34]	Retrospective case–control, 255	First trimester	First-trimester uterine artery PI did not differ between women with GDM and uncomplicated pregnancies, and GDM was not independently associated with UtA PI after adjustment for maternal characteristics.	Retrospective design; small GDM sample; glycemic control data were unavailable.
Tenenbaum-Gavish 2020 [35]	Prospective cohort, 205	11 + 0 to 13 + 6	After adjustment (MoM), pregnancies that later developed GDM had higher UtA-PI. UtA-PI alone had limited predictive performance (AUC 0.63; detection rate 28% at 10% false-positive rate), but combining placental markers with BMI and inflammatory/metabolic biomarkers substantially improved prediction (AUC up to 0.95).	Small sample size, particularly only 20 GDM cases; single-center cohort; prediction model requires external validation before clinical use. The authors describe the findings as preliminary and warranting validation in larger independent cohorts.
Sweeting 2018 [36]	Case–control, 980	11 + 0 to 13 + 6	Women who later developed GDM had lower UtA-PI MoM (1.01 vs. 1.05; *p* = 0.03). Adding placental markers to maternal clinical factors improved prediction from AUC 0.88 to 0.90, with the model performing best for early GDM (AUC 0.96).	Case–control design may overestimate predictive performance; self-reported maternal risk factors (e.g., family history of diabetes) may introduce measurement bias; women with pre-eclampsia were excluded, limiting generalisability; findings require prospective external validation and evaluation using contemporary diagnostic criteria
Tranidou 2024 [37]	Prospective cohort, 4917	11 + 0 to 13 + 6	Higher UtA-PI was associated with subsequent GDM. UtA-PI showed limited predictive ability when used alone. The best prediction was achieved by combining maternal characteristics, obstetric history, and biomarkers.	Single-center study with no external validation; incomplete data on family history and previous GDM may have reduced model accuracy.
Shen 2022 [38]	Prospective cohort, 9372	11 + 0 to 13 + 6	UtA-PI did not independently improve prediction. A model combining maternal characteristics and obstetric history achieved an AUROC of 0.735.	Targeted rather than universal OGTT screening may have underestimated GDM incidence.
Wong, 2019 [18]	Prospective case–control, 155	11 + 0 to 13 + 6, 21 + 0 to 23 + 6	UtA-PI did not differ significantly between pregnancies that developed GDM and controls in either the first or second trimester.	Small sample size with only 31 GDM cases; study included only women at increased risk for GDM, limiting generalizability.

UtA-PI: uterine artery pulsatility index; PI = pulsatility index; GDM = gestational diabetes mellitus; MoM = multiples of median; AUC = area under curve; BMI = body mass index.

## Data Availability

No new data were created or analyzed in this study.

## Abbreviations

The following abbreviations are used in this manuscript:

AAWT	Anterior Abdominal Wall Thickness
AC	Abdominal Circumference
ACOG	American College of Obstetricians and Gynecologists
ADA	American Diabetes Association
ASPRE	Combined Multimarker Screening and Randomized Patient Treatment with Aspirin for Evidence-Based Preeclampsia Prevention
AUC	Area Under Curve
BMI	Body Mass Index
BPD	Biparietal Diameter
CI	Confidence Interval
CRL	Crown-Rump Length
DL	Deep Learning
EFW	Estimated Fetal Weight
FAO	Fetal Abdominal Overgrowth
FAORs	Fetal Abdominal Overgrowth Ratios
FHR	Fetal Heart Rate
FI	Flow Index
FL	Femur Length
GDM	Gestational Diabetes Mellitus
HAPO	Hyperglycemia And Adverse Pregnancy Outcome
HC	Head Circumference
HC:AC	Head Circumference:Abdominal Circumference
IADPSG	International Association of Diabetes in Pregnancy Study Group
MAP	Mean Arterial Pressure
MoM	Multiples Of Median
NIH	National Institutes of Health
NT	Nuchal Translucency
OGTT	Oral Glucose Tolerance Test
OR	Odds Ratio
PAPP-A	Pregnancy-Associated Plasma Protein A
PI	Pulsatility Index
PV	Placental Volume
T1DM	Type 1 Diabetes Mellitus
T2DM	Type 2 Diabetes Mellitus
TCD	Transcerebellar Diameter
UtA-PI	Uterine Artery Pulsatility Index
VFI	Vascularization Flow Index
VI	Vascularization Index
WHO	World Health Organization
β-hCG	β -Human Chorionic Gonadotropin
